# Chronic liver inflammation modifies DNA methylation at the precancerous stage of murine hepatocarcinogenesis

**DOI:** 10.18632/oncotarget.3567

**Published:** 2015-03-14

**Authors:** Evgeniy Stoyanov, Guy Ludwig, Lina Mizrahi, Devorah Olam, Temima Schnitzer-Perlman, Elena Tasika, Gabriele Sass, Gisa Tiegs, Yong Jiang, Ting Nie, James Kohler, Raymond F. Schinazi, Paula M. Vertino, Howard Cedar, Eithan Galun, Daniel Goldenberg

**Affiliations:** ^1^ The Goldyne Savad Institute of Gene Therapy, Hadassah-Hebrew University Medical Center, Jerusalem, Israel; ^2^ Department of Developmental Biology and Cancer Research, Faculty of Medicine, The Hebrew University, Jerusalem, Israel; ^3^ Institute of Experimental Immunology and Hepatology, University Medical Center Hamburg-Eppendorf, Hamburg, Germany; ^4^ Laboratory of Biochemical Pharmacology, Department of Pediatrics, Emory University School of Medicine, and Veterans Affairs Medical Center, Decatur, GA, USA; ^5^ Department of Radiation Oncology and the Winship Cancer Institute, Emory University School of Medicine, Atlanta, GA, USA

**Keywords:** Mdr2 (Abcb4), hepatocellular carcinoma, DNA methylation, mtDNA deletion, 5-hydroxymethylcytosine

## Abstract

Chronic liver inflammation precedes the majority of hepatocellular carcinomas (HCC). Here, we explore the connection between chronic inflammation and DNA methylation in the liver at the late precancerous stages of HCC development in Mdr2^−/−^ (Mdr2/Abcb4-knockout) mice, a model of inflammation-mediated HCC. Using methylated DNA immunoprecipitation followed by hybridization with “CpG islands” (CGIs) microarrays, we found specific CGIs in 76 genes which were hypermethylated in the Mdr2^−/−^ liver compared to age-matched healthy controls. The observed hypermethylation resulted mainly from an age-dependent decrease of methylation of the specific CGIs in control livers with no decrease in mutant mice. Chronic inflammation did not change global levels of DNA methylation in Mdr2^−/−^ liver, but caused a 2-fold decrease of the global 5-hydroxymethylcytosine level in mutants compared to controls. Liver cell fractionation revealed, that the relative hypermethylation of specific CGIs in Mdr2^−/−^ livers affected either hepatocyte, or non-hepatocyte, or both fractions without a correlation between changes of gene methylation and expression. Our findings demonstrate that chronic liver inflammation causes hypermethylation of specific CGIs, which may affect both hepatocytes and non-hepatocyte liver cells. These changes may serve as useful markers of an increased regenerative activity and of a late precancerous stage in the chronically inflamed liver.

## INTRODUCTION

Hepatocellular carcinoma (HCC) typically develops on a background of chronic inflammation induced by viruses or other risk factors that damage the liver and cause compensatory proliferation resulting in hepatocarcinogenesis, a multistep process with accumulation of genetic and epigenetic alterations [1]. Aberrant DNA methylation in tumors has been intensively studied in different cancer types [2-4], including HCC [5-10]. In addition, genome-wide alterations of DNA methylation under precancerous inflammatory conditions were recently demonstrated for several cancer types, including HCC [11, 12]. Aberrant epigenetic changes accumulate in the chronically inflamed liver, preceding and promoting HCC development [13]. Particularly, methylation of specific CGIs is increasing during progression from chronic hepatitis to cirrhosis and to HCC, resulting in the silencing of some tumor suppressor genes [14-17]. However, analysis of the whole liver samples in all cited above studies does not permit identification of a specific cell type, in which aberrant gene methylation and expression take place. In order to explore gene methylation and expression patterns in cell fractions of the chronically inflamed liver, we used the Mdr2-knockout (Mdr2-KO) mice, a well-characterized model of chronic inflammation-mediated HCC [18]. These mutants lack the Mdr2/Abcb4 P-glycoprotein (the murine ortholog of human MDR3) which is responsible for phosphatidylcholine transport across the hepatocyte's canalicular membrane. This causes a dramatic decrease of phospholipids in bile resulting in bile regurgitation into portal tracts [19] and the development of chronic cholestatic hepatitis at an early age (starting from 2 months) and HCC with a high incidence in the adult age (between 12 and 18 months) [18]. This HCC model was widely used to study the molecular mechanisms of inflammation-mediated hepatocarcinogenesis [20-23], HCC transcriptomics [24] and genomics [25, 26]. Previously, using genome-scale gene expression profiling, we revealed multiple aberrantly expressed genes in the liver of Mdr2-KO mice at the late precancerous stage which was characterized by an increased hepatocyte mitosis, steatosis and appearance of dysplastic nodules (Supplementary Figure 1A) [21]. Now, we analyze genome-scale aberrant methylation of CGIs in the liver of these mice at the same stage of chronic liver inflammatory disease and also explore aberrant methylation and expression of several selected genes following liver cell fractionation. To our knowledge, this is the first study exploring the genome-scale liver DNA methylation at the late precancerous stage in a murine model of chronic inflammation-mediated hepatocarcinogenesis.

## RESULTS

### Chronic liver inflammation decreases global level of 5-hydroxymethylcytosine in the liver

**Figure 1:**
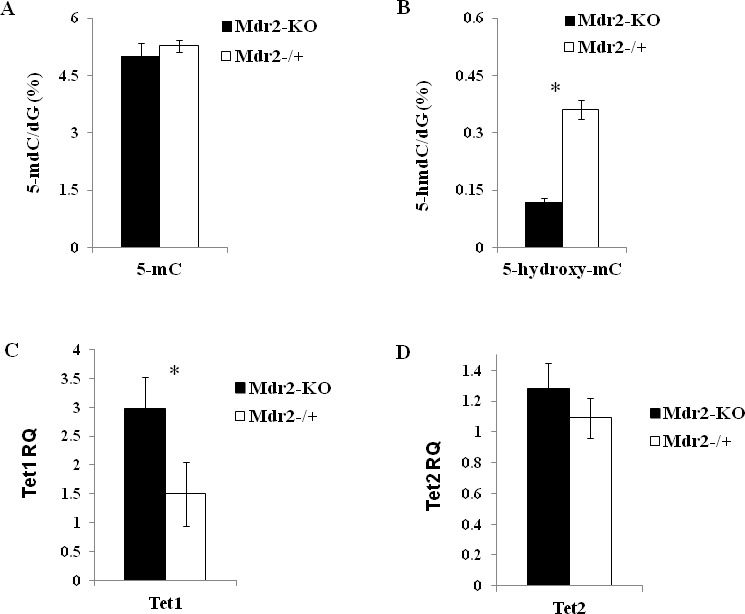
DNA methylation/hydroxymethylation and expression of the Tet genes in Mdr2-KO liver at the late precancerous stage Total levels of 5mC (A) and 5hmC (B) in Mdr2-KO and in the age-matched Mdr2^−/+^ control liver determined by HPLC. (C, D) Expression of the Tet1 (C) and Tet2 (D) genes in the liver Mdr2-KO and Mdr2^−/+^ mice determined by real-time RT-PCR; RQ - relative quantification (normalized to Hprt values). Primers for the Tet genes described in [64]. Three 12-month-old males per group in all experiments; *, p < 0.05.

To determine the effect of chronic liver inflammation on liver DNA methylation, we measured global levels of 5-methylcytosine (5mC) and 5-hydroxymethylcytosine (5hmC) in the liver of Mdr2-KO and control Mdr2^−/+^ mice at the age of 12 months (late precancerous stage for mutants). No difference in the global level of 5mC was found between mutant and control livers when measured by three different methods (Figure 1A; Supplementary Figure 2A,B). Remarkably, a 2.5-fold decrease of the global 5hmC level was detected in mutant livers by the LC-MS/MS method (Figure 1B). Since 5hmC is an intermediate product of 5mC demethylation, its reduced level may indicate a less efficient demethylation process of some CpG sites in the Mdr2-KO liver. Thus, we compared expression of transcripts encoding the Tet proteins, which are responsible for the active demethylation of 5mC by its oxidation to 5hmC [27], in the liver of mutant and control mice at the age of 12 months. The Tet1 expression was significantly increased in the liver of Mdr2-KO compared to Mdr2^−/+^ mice (Figure 1C), while the Tet2 expression was similar in both groups (Figure 1D); the expression of the Tet3 gene in the tested livers was too low for a reliable quantification.

### Chronic liver inflammation causes preferential hypermethylation of specific CpG islands (CGIs)

**Figure 2:**
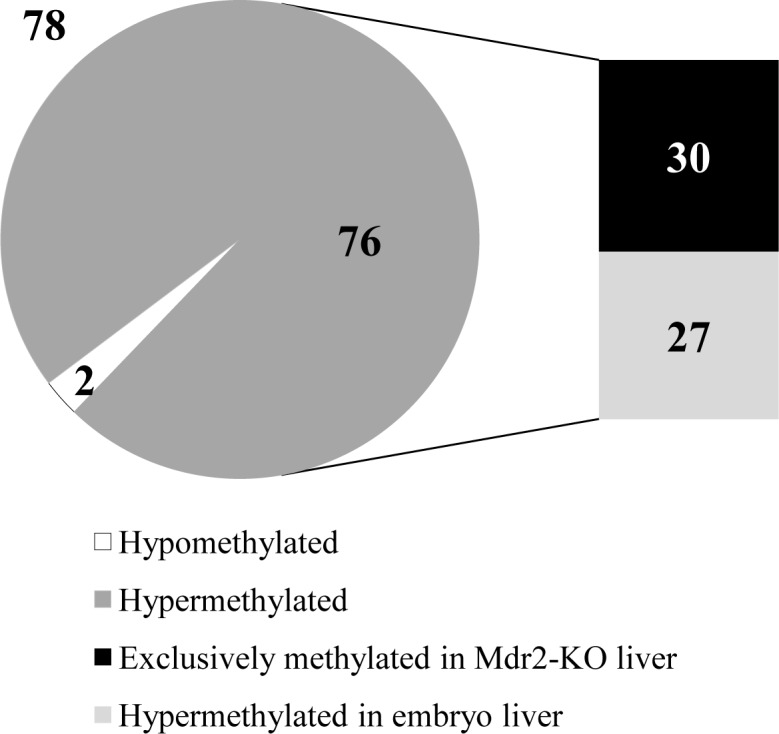
Preferential CGI hypermethylation at the late precancerous stage in Mdr2-KO liver At the late precancerous stage, 78 CGIs were aberrantly methylated in the Mdr2-KO liver compared to Mdr2^−/+^ control. Thirty of these CGIs were specifically hypermethylated in the precancerous Mdr2-KO liver only, while 27 were also hypermethylated at the embryonic stages of liver development. Hybridization to Agilent CGI microarrays of total liver DNA from the 12-month-old Mdr2-KO and Mdr2^−/+^ mice; 3 males per group; Z-score for methylation > 0.7.

To determine the effect of chronic liver inflammation on DNA methylation of CGIs, we used methylated DNA immunoprecipitation (MeDIP) followed by hybridization with CGI microarrays (Agilent; see Materials and Methods) of the liver DNA samples described above. We found that 78 CGIs changed their methylation level significantly: 76 CGIs were hypermethylated and two were hypomethylated in Mdr2-KO mutants compared to controls (Figure 2, Supplementary Table 1 and Supplementary Figure 3). These aberrantly methylated CGIs were distributed among all murine chromosomes with a frequency from 0.2 to 1.8% of all CGIs per chromosome (average frequency of 0.6% of the whole genome CGIs; Supplementary Figure 3A,B). More than half of these CGIs were mapped inside genes and more than 30% were mapped in the promoter regions (Supplementary Figure 3D).

Comparison of the methylation profiles between Mdr2-KO and Mdr2^−/+^ mice and young C57Bl/6 mouse ([28] and H. Cedar, unpublished data) revealed that 30 among the 76 hypermethylated CGIs were specifically methylated only in the tested Mdr2-KO liver: they were methylated neither in the liver at the age of 3, 12 or 18 months, nor in most of the other tested murine tissues of the C57Bl/6 mouse (first 30 CGIs in the Supplementary Table 1). Thus, these 30 hypermethylated CGIs could represent a chronic inflammation-induced methylation signature in this HCC model. In addition, we performed the same MeDIP-CGI methylation analysis for one tumor and its matched non-tumor liver tissue from the 16-month-old Mdr2-KO male (Supplementary Table 1). This tumor had the lowest Mat1a/Mat2a ratio among the tested murine HCCs [24], and thus was expected to have the most aberrant DNA methylation. Additional 27 hypermethylated genes were associated with embryo development, and thus were expected to be hypermethylated in the adult liver (Figure 2). We confirmed hypermethylation of 18 out of the 20 tested CGIs using the MSRE-PCR method (Supplementary Figure 4).

### Absence of the age-dependent demethylation of specific CpG sites in the Mdr2-KO liver

**Figure 3:**
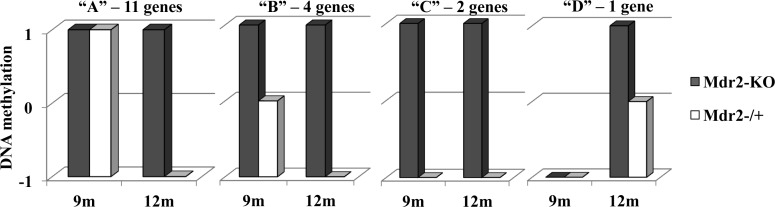
Age-dependent demethylation of aberrantly methylated CGIs DNA methylation (arbitrary units) of the 18 tested CGIs at 9 and 12 months of age in the Mdr2-KO (grey) and Mdr2^−/+^ (white) liver. Fifteen of these 18 genes (“A” and “B” groups) were methylated in 9-month-old mice in both the Mdr2-KO and Mdr2^−/+^ liver, while at the late precancerous stage, in 12-month-old mice, they were specifically demethylated in the control Mdr2^−/+^ liver. An additional two genes (“C”) were hypermethylated at both stages in Mdr2-KO only, and one gene (“D”) was hypermethylated in Mdr2-KO compared to the Mdr2^−/+^ liver at the late precancerous stage only. MSRE-PCR quantified using the ScionImage software and normalized to CryaA; 3 males per group.

To follow the age-dependent DNA methylation dynamics of the group of CGIs which were hypermethylated in Mdr2-KO livers at the age of 12 months, we compared DNA methylation of the 18 CGIs having the higher Z-scores (13 of them were from the group of 30 CGIs mentioned above) at 9 and 12 months of age in mutant and control livers using MSRE-PCR (Figure 3, Supplementary Table 2). We found that only one of the tested CGIs was not methylated in the Mdr2-KO liver at the age of 9 months and became methylated at the age of 12 months. All other tested CGIs were methylated in the Mdr2-KO liver at both ages. However, in the control Mdr2^−/+^ liver, 15 of these CGIs were methylated at the age of 9 months (11 were highly methylated and 4 were methylated), while they became unmethylated at the age of 12 months. Thus, in most tested cases, hypermethylation of the specific CGIs in the Mdr2-KO compared to the control liver resulted from an age-dependent demethylation of these sites in the control, but not in the mutant liver. Interestingly, this defective age-dependent demethylation of a specific set of CpG sites correlates with the reduced level of 5hmC in the Mdr2-KO liver.

**Figure 4:**
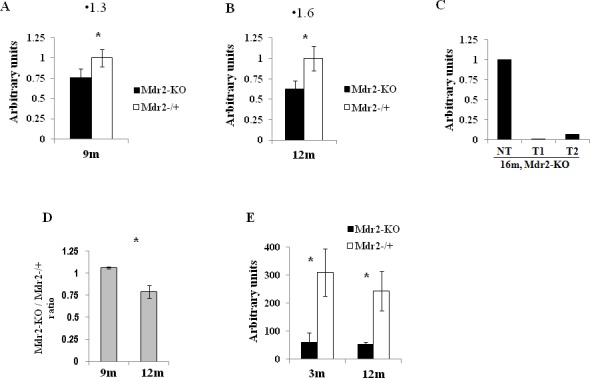
Markers of liver compensatory regeneration in Mdr2-KO mice at the late precancerous stage Decreased incidence of the large mitochondrial deletions in the chronically inflamed liver of the Mdr2-KO compared to Mdr2^−/+^ mice at the age of 9 months (A) and 12 months (B), as well as in tumors compared to the matched non-tumorous liver tissue of 16-month-old Mdr2-KO mice (C); normalization to total mtDNA. (D) Relative total mtDNA levels (ratio of Mdr2-KO to Mdr2^−/+^) in the liver of 9- and 12-month-old mice; semi-quantitative RT-PCR, quantified with the ScionImage software; *, p<0.05. (E) Decreased incidence of binuclear hepatocytes in the liver of Mdr2-KO compared to Mdr2^−/+^ mice both at the early and late precancerous stages; haematoxylin staining of paraffin-embedded liver tissues, 10 fields/mouse at a magnification of ×100 were counted, three mice per group, *, p<0.002.

**Figure 5:**
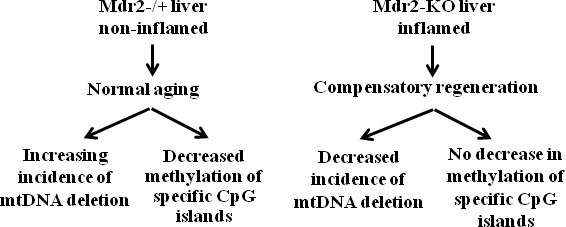
Schematic representation of the events which take place in the chronically inflamed liver of Mdr2-KO mice at the precancerous stages The scheme summarizes results presented in figures 1 to 4.

The defective age-dependent demethylation of specific CpG sites in the Mdr2-KO liver could stem from a partially de-differentiated, proliferative state of hepatocytes and cholangiocytes due to the extensive compensatory regeneration of the chronically inflamed mutant liver. This hypothesis is supported by our previous finding on the decreased level in 12-month-old Mdr2-KO compared to the Mdr2^−/+^ liver of a large mitochondrial DNA (mtDNA) deletion whose incidence is known to be increased either with age or following oxidative stress [21]. Here, we confirm the decreased level of mtDNA deletion in 12-month-old Mdr2-KO compared to the Mdr2^−/+^ liver, and demonstrate that a similar decrease, albeit less significant, takes place already in 9-month-old mice (Figure 4A,B). For comparison, in tumors of the 16-month-old Mdr2-KO mice, the level of mtDNA deletion was significantly lower than in the matched non-tumor liver tissue (Figure 4C). Remarkably, the total level of mtDNA in the liver of Mdr2-KO mice was significantly reduced at 12 months compared to 9 months of age (Figure 4D). A reduced incidence of binuclear hepatocytes, a known marker of the regenerating liver [29], was found both in the 3- and 12-month-old Mdr2-KO compared to control mice (Figure 4E). These data are consistent with the high proliferative activity of hepatocytes and cholangiocytes in 12-month-old Mdr2-KO mice (which can be interpreted as a “younger age” of these cells), due to a prolonged, chronic inflammation-induced, extensive compensatory regeneration of the mutant liver (Figure 5).

### Aberrant methylation of specific CGIs in the chronically inflamed liver at the late precancerous stage affected mostly low-expressed genes

Previously we reported a list of genes which were aberrantly expressed in Mdr2-KO compared to control liver in 3- and 12-month-old mice [21]. Now, we compared aberrantly methylated and aberrantly expressed genes in 12-month-old mice (Supplementary Figure 5). There were 8,677 genes present both on the expression (Affymetrix) and CGI methylation (Agilent) microarrays. Among 78 aberrantly methylated genes, 37 were present on the expression microarray, and among 424 aberrantly expressed genes, 252 were present on the methylation microarray. However, none of these genes was simultaneously aberrantly expressed and aberrantly methylated in the Mdr2-KO liver, at least, on the detection level of these microarrays (Supplementary Figure 5).

**Figure 6:**
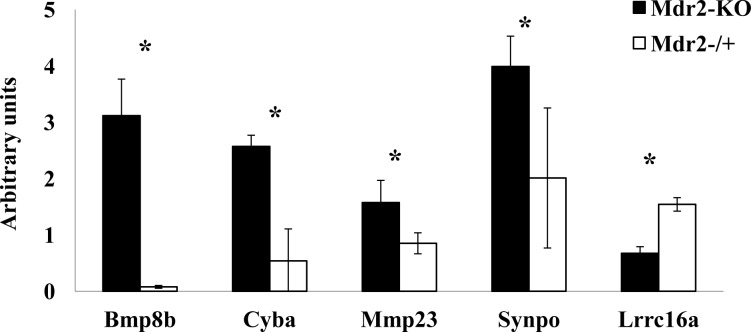
Direct correlation between expression and methylation of selected aberrantly methylated genes Hypermethylated genes Bmp8b, Cyba, Mmp23, Synpo were up-regulated, while hypomethylated gene Lrrc16a was down-regulated in the Mdr2-KO compared to the Mdr2^−/+^ liver at the late precancerous stage (three 12-month-old males per group). Semi-quantitative (for Cyba, Mmp23, Synpo and Lrrc16a) or real-time (for Bmp8b) RT-PCR normalized to Gapdh and quantified using the ScionImage software, *, p<0.05.

We then checked the gene expression levels of the 18 genes which were aberrantly methylated in Mdr2-KO compared to control liver at the late precancerous stage (16 hyper- and two hypomethylated; 11 of them were from the list of 30 CGIs hypermethylated exclusively in the Mdr2-KO livers) using RT-PCR. We found that 13 of the 18 tested genes were expressed in the murine liver; however, their expression levels were low. Only five from the 13 expressed genes changed their expression level in the Mdr2-KO compared to control liver: the hypermethylated genes Bmp8b, Cyba, Mmp23, and Synpo were up-regulated, while the hypomethylated gene Lrrc16a was down-regulated (Figure 6, Supplementary Table 3). Thus, the most aberrantly methylated genes in the Mdr2-KO liver were either not expressed, or did not change their expression level; in the aberrantly methylated and aberrantly expressed genes, there was a direct correlation between changes of their expression and methylation levels.

### Testing correlation between gene methylation and expression in cellular fractions of the Mdr2-KO liver

**Figure 7:**
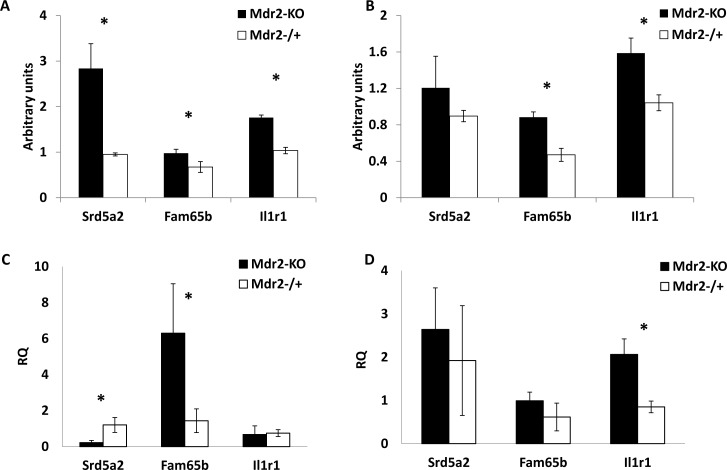
Methylation and expression of selected genes in liver cell fractions Genes Srd5a2, Fam65b and Il1r1 which were hypermethylated in total liver DNA of *Mdr2*-KO mice at the late precancerous stage (12-month-old mice), were now tested in hepatocyte and non-hepatocyte fractions of Mdr2-KO and Mdr2^−/+^ mice at the same age. (A,B) gene methylation; (C,D) gene expression; (A,C) hepatocyte fraction; (B,D) non-hepatocyte fraction. Srd5a2 was hypermethylated in Mdr2-KO hepatocytes (A), while Fam65b and Il1r1 were hypermethylated in both Mdr2-KO hepatocyte (A) and non-hepatocyte fractions (B); MSRE-PCR, relative to Mdr2^−/+^ normalized to CryaA and quantified using ScionImage; three 12-month-old males per group; *, p<0.05. (C) Srd5a2 down-regulated, Fam65b up-regulated and Il1r1 unchanged in Mdr2-KO hepatocytes; (D) Il1r1 up-regulated, Srd5a2 and Fam65b unchanged in Mdr2-KO non-hepatocyte cells. Real-time RT-PCR, relative to Mdr2^−/+^, normalized to Gapdh values; five 12-month-old males per group; *, p<0.02.

In order to explore whether aberrant gene methylation and expression took place in hepatocytes or other liver cells, we fractionated the livers of the Mdr2-KO and control age- and sex-matched Mdr2^−/+^ mice into hepatocyte and non-hepatocyte cell fractions. We compared methylation (Figure 7A,B, Supplementary Figure 6) and expression (Figure 7C,D) levels of the genes Fam65b, Il1r1 and Srd5a2 in liver cell fractions of mutant and control mice. In the whole liver extracts, the appropriate CGIs of these three genes were hypermethylated (Figure 3 and Supplementary Table 2); however, all three genes were not aberrantly expressed (Supplementary Figure 7). These genes were chosen due to their high fold-change of the methylation Z-score in the mutant liver (Fam65b and Srd5a2) and their important roles in inflammation (Il1r1) and HCC development (Srd5a2). The tested CGIs of these three genes had different methylation patterns in liver cell fractions: Srd5a2 was hypermethylated mainly in hepatocytes, Fam65b – mainly in non-hepatocytes, while Il1r1 was similarly hypermethylated in both cell fractions (Figure 7A,B and Supplementary Figure 6). A strong hypermethylation of Srd5a2 in hepatocytes resulted in a significant decrease of its expression (Figure 7C), while its weak hypermethylation in non-hepatocytes (which could be a result of a minor contamination with hepatocytes) did not affect gene expression (Figure 7D). For Fam65b, a stronger hypermethylation of the non-hepatocyte fraction did not affect gene expression, while a weaker hypermethylation of the hepatocyte fraction resulted in a significant increase of its expression. In the case of the Il1r1 gene, a similar efficient hypermethylation in both fractions resulted in a significant increase of its expression in non-hepatocyte cells, while it did not affect its expression in hepatocytes (Figure 7C,D). These results demonstrate that chronic inflammation-induced relative hypermethylation of specific CGIs at the late precancerous stage may affect both hepatocyte and non-parenchymal liver cells, resulting in unpredictable changes of expression of the appropriate genes in the affected cell fractions.

### At the tumor stage, the tested CGIs were preferentially methylated either in tumors or in non-tumor tissue of the Mdr2-KO liver

We demonstrated, using MeDIP followed by hybridization with the CGI microarray, that most CGIs that were hypermethylated in the Mdr2-KO liver at the age of 12 months were also methylated in a tested HCC tumor at the age of 16 months (Supplementary Table 1). In order to expand our knowledge on the methylation status of these CGIs in tumors, we compared the methylation levels between the six selected CGIs in seven additional HCC tumors (see representative pictures of their morphology and histology in Supplementary Figure 1B,C) and their matched non-tumor liver tissues of 16-month-old Mdr2-KO mice using MSRE-PCR (Supplementary Table 4; Supplementary Figure 8). In most tested cases, these CGIs were methylated both in tumors and in non-tumor liver tissues (Supplementary Figure 8); however, they demonstrated different degrees of methylation in these two tissue types. The genes Fam65b and Il1r1 were methylated preferentially in the non-tumor liver tissue, whereas the genes Synpo and Tspan9 were methylated preferentially in tumors; the gene Srd5a2 in each half of the tested tumors was preferentially methylated in either the non-tumor tissue or in tumors (Supplementary Figure 8).

## DISCUSSION

In this study, we have found preferential relative (compared to the age-matched controls) hypermethylation of specific CGIs in the Mdr2-KO liver at the late precancerous stage of chronic hepatitis. The affected CGIs mapped mostly inside genes; the methylation of 30 of them was highly specific for this specific stage and for this specific HCC model. The detected changes of DNA methylation could be explained by either age- or inflammation-dependent processes. Chronic inflammation is known to induce changes in epigenetic machineries, including disruption of tissue- and cell-specific DNA methylation patterns, resulting both in hyper- and hypo-methylation of specific CpG sites [30]. These changes in turn may contribute to the exacerbation of chronic inflammation, thus producing a positive feedback loop between inflammatory and epigenetic changes which can promote proliferation and oncogenic transformation [31]. Cancer cells exhibit increased methylation at multiple gene-associated CGIs with a concomitant widespread decrease of DNA methylation outside CGIs [4]. Methylation of specific CGIs is gradually increased during multistep carcinogenesis and frequently the same CGIs have been found to be increasingly methylated with age in normal tissues [32, 33]. In hepatocarcinogenesis, methylation of multiple CGIs is increasing during progression from chronic hepatitis to cirrhosis and to HCC [14-17]. Thus, our finding of preferential hypermethylation of CGIs in the chronically inflamed liver of Mdr2-KO mice at the late precancerous stage is in agreement with previously published human data.

Age-dependent changes of DNA methylation in the murine liver were shown to be progressive, tissue-specific and included both hypo- and hyper-methylation [34]. In agreement with these data, we also detected a set of CGIs which were hypo-methylated in the control Mdr2^−/+^ liver at the age of 12 months compared to the age of 9 months.

The absence of this age-dependent hypomethylation in the Mdr2-KO liver could be explained by an age-dependent active demethylation of these CGIs in the control, but not in the mutant liver. The reduced 5hmC level in the Mdr2-KO liver may reflect, in part, the reduced active demethylation of multiple CpG sites; the total number of such sites in the genome could be significantly higher than the number of the specific CGIs detected in our study. The level of 5hmC in the liver is known to be decreased in cancer and at the pre-cancerous stage [35], including human liver cancer [36] and chemically induced hepatocarcinogenesis in mice [37], as well as in rodent liver subjected to chronic [38] or sub-chronic [39] genotoxic treatments. We are not aware of studies exploring the 5hmC dynamics during liver regeneration; however, the recent finding of the reduced 5hmC level during regeneration of zebrafish fin [40] supports the negative correlation between cell proliferation and 5hmC level. In the healthy liver, this negative correlation is supported by findings of the reduced 5hmC level in the fetal compared to the adult human liver [41] and in the young compared to the old mouse liver [42]. Thus, the decreased level of 5hmC, together with the defective age-dependent demethylation of specific CGIs, the decreased levels of mtDNA deletion and of total mtDNA, and the reduced number of binuclear hepatocytes in the aged Mdr2-KO mice, support our hypothesis of a partially de-differentiated, proliferative state of their hepatocytes and cholangiocytes due to an extensive and prolonged compensatory regeneration of the chronically inflamed mutant liver.

The exact molecular mechanisms responsible for the decreased 5hmC level in the Mdr2-KO liver are yet to be defined. In different cancer types, including HCC, a decreased level of 5hmC was shown to be caused by either reduced expression of the TET proteins or by inhibition of their activity by toxic metabolite α-hydroxyglutarate which is produced by gain-on-function mutant isocitrate dehydrogenases IDH1 or IDH2 [27, 36]. Remarkably, a decreased 5hmC level in tumors is often associated with a concomitant reduction of the levels of all three TET transcripts – TET1, TET2 and TET3 [36]. In human HCC, a decreased 5hmC was associated with disease progression through down-regulation of TET1 [37], while high 5hmC and IDH2 levels were associated with a favorable prognosis following tumor resection [43]. In contrast to these data, in the Mdr2-KO liver at the late precancerous stage, a decreased 5hmC level was associated with significantly increased levels of the Tet1 (Figure 1C) and Idh2 (our data from microarray gene expression profiling [21]) transcripts (Tet2 and Idh1 levels were not changed; Tet3 was undetectable). Recently, it was shown that succinate dehydrogenase (SDH) deficiency decreases the 5hmC level in gastrointestinal tumors by a metabolic inhibition of TET2 activity [44]. However, expression of the Sdha, Sdhb, Sdhc, and Sdhd genes was similar in Mdr2-KO and matched control liver (our microarray data [21]). Mutations in the Sdh genes as well as gain-on-function mutations in the Idh1 or Idh2 genes should be excluded in this case, due to the absence of clonality in the non-tumor liver tissue, in contrast to tumors. Further studies are required to reveal the molecular mechanisms responsible for the reduced 5hmC level in the chronically inflamed Mdr2-KO liver.

Recent studies of genome-scale DNA methylation revealed a more complicated correlation between the promoter's CGI methylation and gene expression than the previously suggested up-regulation of hypomethylated and down-regulation of hypermethylated genes [45]. This reversed correlation between promoter CGI methylation and gene expression is observed mostly for high-CGI dense promoters, whereas other promoters do not exhibit such correlation in liver cancer [9]. Analysis of the whole-genome methylation demonstrates that the repressive effect of promoter methylation on gene expression is clear only on genes with a very high DNA methylation level, and that the gene body methylation is a better indicator of gene expression than promoter methylation [45]. The small overlap of differentially methylated and differentially expressed genes can also be explained by the fact that DNA methylation in different cancer types affects mostly low-expressed or non-expressed genes [46]. Similarly, in the Mdr2-KO liver at the precancerous stage, most aberrantly methylated genes had either a low or an undetectable expression level, and none of them had a reverse correlation between DNA methylation and gene expression levels.

Analysis of gene regulation in the liver is complicated by the organ's heterogeneity: different populations of resident cells, especially in the inflamed liver, could also play a role in the absence of a correlation between gene methylation and expression. A small population of cells in a tissue contributes little to the whole liver expression or methylation level when the expression or methylation of a gene in this population is decreased; however, it may contribute significantly when the expression or methylation of a gene in it is increased, especially in a case of low or undetectable level of whole liver expression or methylation. We applied liver fractionation which produces two cell fractions: one homogeneous (mostly hepatocytes) and one heterogeneous (other liver cells) to explore three genes which were hypermethylated and similarly expressed in the whole extracts of the Mdr2-KO compared to control livers. We could not find an obvious correlation between the expression and CGI methylation of these genes in liver cell fractions. In hepatocytes, all of their CGIs were hypermethylated compared to controls; however, the expression of Srd5a2 was decreased, while the expression of Fam65b was increased and the Il1r1 expression was unchanged. In the non-hepatocyte fraction, both Fam65b and Il1r1 were hypermethylated, but only Il1r1 increased its expression. The liver's non-hepatocyte fraction comprises many cell types, and thus analysis of gene expression and methylation in it is complicated, similarly to the whole liver.

Previously, we demonstrated that the Mdr2-KO mouse is a relevant model for human HCC in terms of a similarity of the aberrant gene expression patterns in murine and human liver tumors [24]. Now, we demonstrate the relevance of this HCC model also at the late precancerous stage. The absolute level of 5hmC in the control murine liver and its decrease in the Mdr2-KO liver at the precancerous stage is similar to what was observed at the same stages in patients [35]. A decrease of the total mtDNA level was observed in human HCC tumors [47], while a reduced frequency of the large mtDNA deletion was detected in both liver cirrhosis and HCC [48, 49].

Although mice and humans share a common pattern of epigenetic changes during hepatocarcinogenesis, the specific aberrantly methylated CpG sites are expected to be mainly species-specific. Thus, fundamental differences in promoter CGI methylation have been revealed between three human cancers and their appropriate murine models [50]. In addition, the recently found novel “epigenetic clock” - a set of 353 CpG sites whose methylation for multiple healthy human tissues increases with age - for blood cells, is well applicable to chimpanzees, but poorly – to gorillas [51]. Nevertheless, some genes with hypermethylated CGIs in the Mdr2-KO model were also aberrantly methylated in human HCC. The SRD5A2 gene was hypermethylated and down-regulated in early stages of human HCC [52] and was even used in optimal blood tests for HCC detection in HCV-infected patients [53]. Genes CELSR1 and ST8SIA3 were also hypermethylated in human HCC [17, 54]. Genes Fam65b and Il1r1 are known to play a role in cancer, and liver-, or inflammation-associated diseases. Fam65b protein binds the small GTPase RhoA and represses its activity by decreasing its GTP loading, negatively regulating by this cell adhesion, morphological polarization, and migration [55]. Interleukin-1 and its receptor encoded by the IL1R1 gene regulate progression from liver injury to fibrosis [56]; IL1R1 polymorphism is associated with the risk for inflammatory bowel disease [57]. Hypermethylation of these genes, together with others identified in our study, may serve as a marker for the late precancerous stage of the chronically inflamed liver reflecting such processes as partial hepatocyte dedifferentiation due to the compensatory regeneration and formation of a pro-tumorigenic state of the liver.

We demonstrated in the chronically inflamed liver at the late precancerous stage the appearance of highly specific aberrant methylation events (mostly, hypermethylation) of a set of CGIs. This aberrant methylation affected both hepatocyte and non-hepatocyte cells and in some cases resulted also in changes of expression of the affected genes. Some of the changes that we have found in the Mdr2-KO HCC model also take place in human hepatocarcinogenesis either at the precancerous or at the early HCC stage. Thus, our findings of aberrant DNA methylation of specific CGIs may have a diagnostic significance for the late precancerous or early cancerous stages of HCC development in the chronically inflamed liver.

## MATERIALS AND METHODS

### Mice

All animal experiments were performed according to national regulations and guidelines of the Institutional Animal Welfare Committee (NIH approval number OPRR-A01-5011). The FVB.129P2-Abcb4^tm1Bor^ (Mdr2-KO) and wild type FVB/NJ mice were purchased from the Jackson Laboratory (Bar Harbor, ME); Mdr2^−/+^ heterozygotes were produced by breeding of the Mdr2-KO and FVB/NJ mice and used as controls. Mice obtained a regular diet and drinking water ad libitum and under controlled conditions (22°C, 55% humidity, and 12-hour day-night rhythm). Only males were used in this study. Harvesting of mouse liver tissue was done as described previously [21].

### Fractionation of liver cells

Primary hepatocytes were isolated as previously described [58]. Briefly, the livers were perfused with Liberase and live hepatocytes were isolated using precipitation by gravitation force and then by centrifugation in Percoll gradient. In addition, non-hepatocyte fraction was collected. DNA was purified using Wizard Genomic DNA Purification kit (Promega, WI, USA). RNA was purified using Trizol as described in Materials & Methods and treated with Ambion TURBO DNase (Life Technologies, CA, USA); the cDNA qScript Synthesis kit (Quanta, Biosciences, Gothenburg, Sweden) was used according to the manufacturer's instructions. Both fractions were checked for hepatocyte and T cell specific markers (Albumin and T-cell receptor, respectively; not shown).

### Purification of total liver DNA and RNA

Total liver DNA and RNA was extracted from frozen liver tissue specimens as previously described [24].

### Deletions in mitochondrial DNA

The D1 deletion in mitochondrial DNA was detected in total liver DNA by semi-qPCR as previously described [21].

### cDNA synthesis and semi-quantitative RT-PCR

The cDNA obtained from one microgram of total liver RNA was used for semi-qRT-PCR as previously described [21].

### Real-time RT-PCR

Real-time RT-PCR was run in triplicates using the PerfeCTa SYBR Green Fast mix ROX (Quanta Biosciences, Gothenburg, Sweden) or TaqMan Fast Universal PCR Master Mix (AB Applied Biosystems, CA, USA) primers and probe sets, on the Fast Real-Time PCR System 7900HT (Applied Biosystems, CA, USA). Threshold cycle numbers (C_t_) were determined with Sequence Detector Software (version 1.6) and transformed using the ΔΔC_t_ method as described by the manufacturer. The relative quantification values for each gene were normalized against the endogenous “housekeeping” gene Arl6ip1 or Hprt.

### Global DNA methylation measurements

The global levels of 5-hydroxymethylcytosine (5hmC) and 5-methycytosine (5mC) were measured by liquid chromatography tandem mass spectrometry (LC-MS/MS) method on a Dionex Ultimate 3000 HPLC system interfaced with an AB SCIEX API 5000 Triple quadruple mass spectrometer as described previously [59] with the minor change of using the nucleoside analog Lamivudine as an internal standard. In addition, global methylation level of 5-mC was assessed using the MethylFlash™ Methylated DNA Quantification Kit (Epigentek, Brooklyn, NY) according to the manufacturer's protocol and by measuring the methylation level of the B1 SINE element [60]. Liver genomic DNA was bisulfite-treated using EZ DNA Methylation Direct kit (Zymo Research, CA, USA) following the manufacturer protocol. PCR on bisulfite-treated DNA was performed in similarity with sqRT-PCR reactions (detailed in semi-quantitative RT-PCR method) with the 0.1M primers designed with the assistance of the online tool MethPrimer [61].

### Methylated DNA immunoprecipitation (MeDIP) followed by hybridization to CpG island microarray

Enrichment of total liver DNA with methylated DNA fraction was performed as previously described [62]. The anti 5-methylcytosine antibody was provided by Prof. H. Cedar. The enrichment of the precipitated fraction in methylated DNA was measured by real-time RT-PCR of the methylated CryaA gene and unmethylated Aprt gene on both DNA fractions (“methylated DNA enriched” and “input”). Both total (“input”) and methylated DNA fractions were labeled with either Cy3 or Cy5 and hybridized to the high-density two-color Mouse CpG island microarray G4811A (Agilent Technologies, Santa Clara, CA, USA) according to the manufacturer's instructions. The Agilent G4811A array (printed using 60-mer SurePrint technology) originally designed based on the UCSC genome mm8, each contains 95,830 probes that tile through each of the 15,342 CpG islands. Each probe on the array was identified by its location on the genome and its associated gene(s) based on UCSC annotations. The bioinformatics analysis of the raw array data was performed as previously described [63]. To increase statistical significance of the obtained results, all CGIs whose delta Z-score values were lower than 0.7 were excluded from the resulting tables. Thus, all methylated CGIs in this study (having the mark “1”) have a high and a highly statistically significant methylation level. The DNA methylation data obtained from microarrays can be accessed from the GEO-NCBI database repository (GSE64097).

### Methylation-sensitive restriction enzymes (MSRE) PCR

One microgram of each DNA sample was digested in a 80μl reaction volume by HpaII or MspI endonucleases according to the manufacturer's instruction (NEB, MA, USA). The quality of digestion was assessed by gel electrophoresis; two microliters of the reaction mixture were used as a template for PCR. The intensities of the resulting bands in a gel were compared between experimental groups following normalization to the intensity of a PCR product of the control CryaA gene which does not contain HpaII/MspI recognition sites.

### Statistical analysis

All parameters were evaluated by the two-tailed t-test. A “p” value of 0.05 or less was considered significant. The data are expressed as a mean ± standard deviation (SD).

## SUPPLEMENTARY MATERIAL, FIGURES, TABLES



## Abbreviations

5hmC: 5-hydroxymethylcytosine
5mC: 5-methylcytosine
Ahcy: S-adenosylhomocysteine hydrolase
Bmp8b: bone morphogenetic protein 8b
Celsr1: cadherin, EGF LAG seven-pass G-type receptor 1
CryaA: Crystalline A
Cyba: cytochrome β-245, alpha polypeptide
Fam65b: family with sequence similarity 65, member B
FVB: FVB/NJ
HCC: hepatocellular carcinoma
IDH: isocitrate dehydrogenase
Il1r1: interleukin 1 receptor, type I
LC-MS: Liquid chromatography–mass spectrometry
Mat: Methionine Adenosyl-Transferase
Mdr2: mouse gene encoding the multidrug resistance-2 (Abcb4) protein
MeDIP: methylated DNA immunoprecipitation
Mmp23: matrix metallopeptidase 23
MSRE: Methyl-Sensitive Restriction-Enzyme-dependent
mtDNA: mitochondrial DNA
PCR: polymerase chain reaction
RT-PCR: reverse transcription polymerase chain reaction
SAM: S-adenosylmethionine
SDH: succinate dehydrogenase
SINE: Short Interspersed *Element*
Srd5a2: steroid-5-α-reductase, α polypeptide 2
St8sia3: ST8 alpha-N-acetyl-neuraminide alpha-2,8-sialyltransferase 3
TET: Ten-Eleven Translocation proteins
WT: wild type
