# Supplementary material for: Chronic liver inflammation modifies DNA methylation at the precancerous stage of murine hepatocarcinogenesis
Source: Oncotarget. 2015 Mar 14;6(13):11047–60. doi: 10.18632/oncotarget.3567 (PMC4484438; doi:10.18632/oncotarget.3567)
Supplement: Supplementary file 1 [file oncotarget-06-11047-s001.pdf]

Chronic liver inflammation modifies DNA methylation at the precancerous stage of murine hepatocarcinogenesis

SUPPLEMENTARY MATERIALS AND METHODS

Mice. Mice obtained a regular diet and drinking water *ad libitum* and under controlled conditions (22°C, 55% humidity, and 12-hour day-night rhythm). Only males were used in this study.

Fractionation of liver cells. Briefly, the livers were perfused with Liberase and live hepatocytes were isolated using precipitation by gravitation force and then by centrifugation in Percoll gradient. In addition, non-hepatocyte fraction was collected. DNA was purificated using Wizard Genomic DNA Purification kit (Promega, WI, USA). RNA was purificated using Trizol as described in Materials & Methods and treated with Ambion TURBO DNase (Life Technologies, CA, USA); the cDNA qScript Synthesis kit (Quanta, Biosciences, Gothenburg, Sweden) was used according to the manufacturer's instructions. Both fractions were checked for hepatocyte and T cell specific markers (Albumin and T-cell receptor, respectively; not shown).

Real-time RT-PCR. Threshold cycle numbers (C_t) were determined with Sequence Detector Software (version 1.6) and transformed using the $\Delta\Delta C_t$ method as described by the manufacturer. The relative quantification values for each gene were normalized against the endogenous "housekeeping" gene Arl6ip1 or Hprt.

Global DNA methylation measurements. Liver genomic DNA was bisulfite-treated using EZ DNA Methylation Direct kit (Zymo Research, CA, USA) following the manufacturer protocol. PCR on bisulfite-treated DNA was performed in similarity with sqRT-PCR reactions (detailed in Semi-quantitative PCR Method) with the 0.1M primers designed with the assistance of the online tool MethPrimer [1]. Quantification of the 5mdC and 5hmdC global levels by liquid chromatography tandem mass spectrometry (LC-MS/MS) was performed on a Dionex Ultimate 3000 HPLC system interfaced with an AB SCIEX API 5000 Triple quadruple mass spectrometer

as described before [2] with the minor change of using the nucleoside analog Lamivudine as an internal standard.

Methylated DNA immunoprecipitation followed by hybridization to CpG island microarray. The enrichment of the precipitated fraction in methylated DNA was measured by real-time RT-PCR of the methylated CryaA gene and unmethylated Aprt gene on both DNA fractions ("methylated DNA enriched" and "input"). The Agilent G4811A array (printed using 60-mer SurePrint technology) originally designed based on the UCSC genome mm8, each contains 95,830 probes that tile through each of the 15,342 CpG islands. Each probe on the array was identified by its location on the genome and its associated gene(s) based on UCSC annotations. To increase statistical significance of the obtained results, all CGIs whose delta Z-score values were lower than 0.7 were excluded from the resulting tables. Thus, all methylated CGIs in this study (having the mark “1”) have a high and a highly statistically significant methylation level.

Methylation-sensitive restriction enzymes (MSRE) PCR. One microgram of each DNA sample was digested in a 80µl reaction volume by HpaII or MspI endonucleases according to the manufacturer’s instruction (NEB, MA, USA). The quality of digestion was assessed by gel electrophoresis; two microliters of the reaction mixture were used as a template for PCR. The intensities of the resulting bands in a gel were compared between experimental groups following normalization to the intensity of a PCR product of the control CryaA gene which does not contain HpaII/MspI recognition sites.

References

1. Li LC, Dahiya R. *MethPrimer: designing primers for methylation PCRs.* Bioinformatics **2002**, 18(11):1427-31.
2. Jin SG, Jiang Y, Qiu R, Rauch TA, Wang Y, Schackert G, Krex D, Lu Q, Pfeifer GP. *5-Hydroxymethylcytosine is strongly depleted in human cancers but its levels do not correlate with IDH1 mutations.* Cancer Res **2011**, 71(24):7360-5.

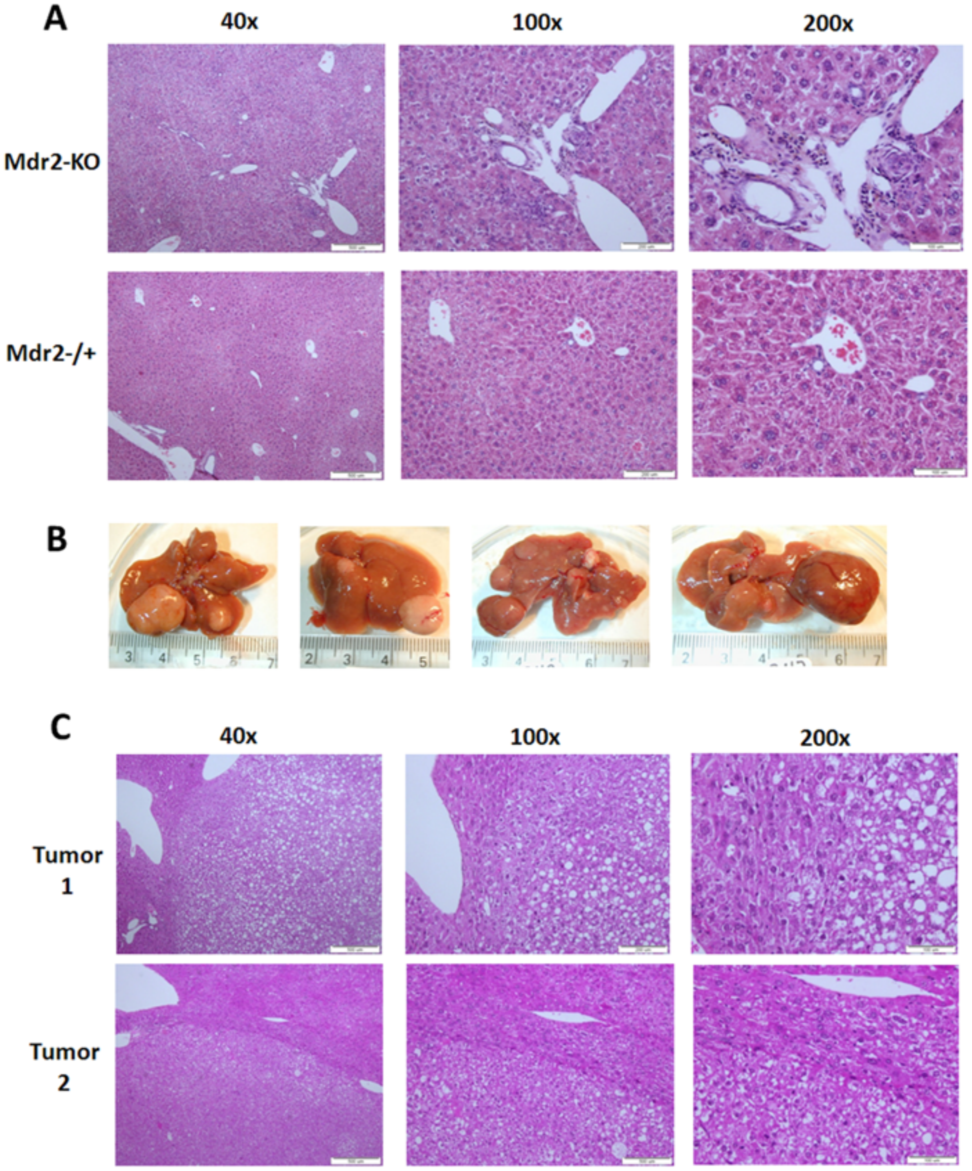

Supplementary Figure 1: Liver of Mdr2-KO mice at the late precancerous and cancer stages. (A) Portal inflammation in the liver of 12-month-old Mdr2-KO compared to control healthy Mdr2$^{-/+}$ male mouse (H & E staining, magnifications 40x, 100x, 200x). (B) Morphology of liver tumors of the 16-month-old Mdr2-KO male mice. (C) Histology of liver tumors of the 16-month-old Mdr2-KO male mice (H & E staining; magnifications 40x, 100x, 200x).

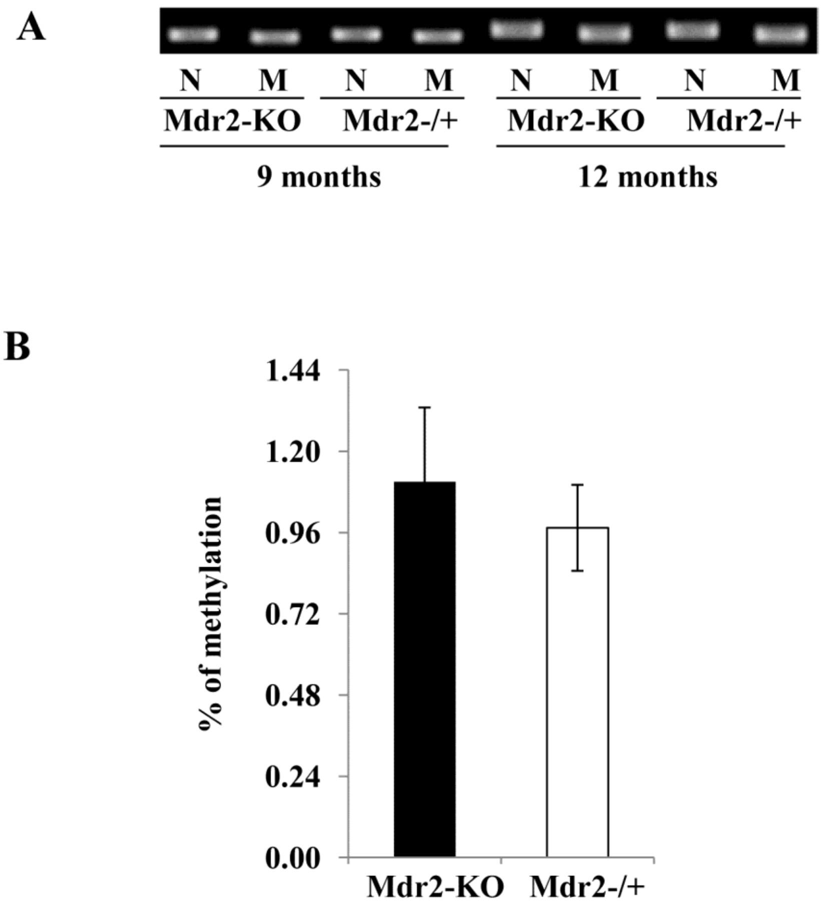

Supplementary Figure 2: Total DNA methylation at the precancerous stages. Similar methylation levels of total liver DNA in Mdr2-KO and control mice, as measured by (A) RT-PCR of the SINE elements of 9- and 12-month-old mice and by (B) Methyl-Flash Colorimetric assay at the age of 12 months (representative gel); “N” - non-methylated, “M” - methylated sequence primers; 3 mice per group; p>0.05 for all cases.

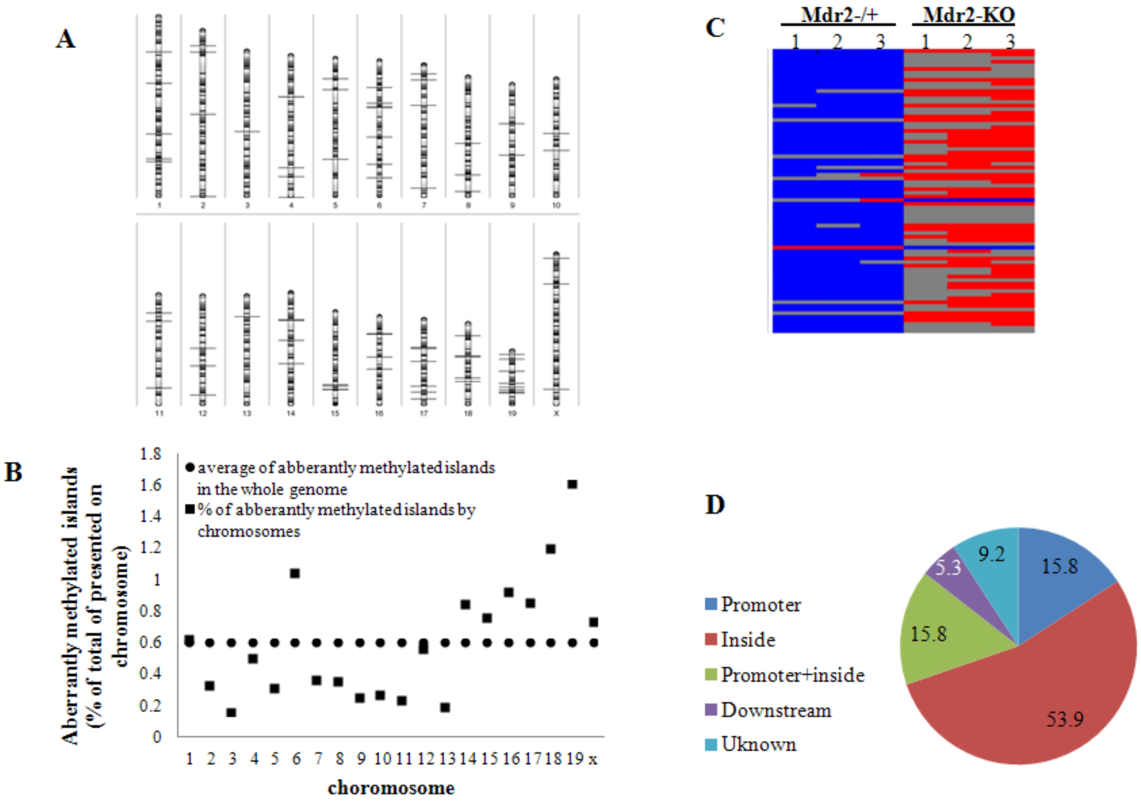

Supplementary Figure 3: Characterization of the CGIs which were aberrantly methylated in Mdr2-KO liver at the late precancerous stage. Seventy eight aberrantly methylated CGIs represented as: a genome map (A), a distribution relative to the expected CGI methylation in chromosomes (B); a distribution of microarray triplicates (C); and a location relative to a proximal gene (D).

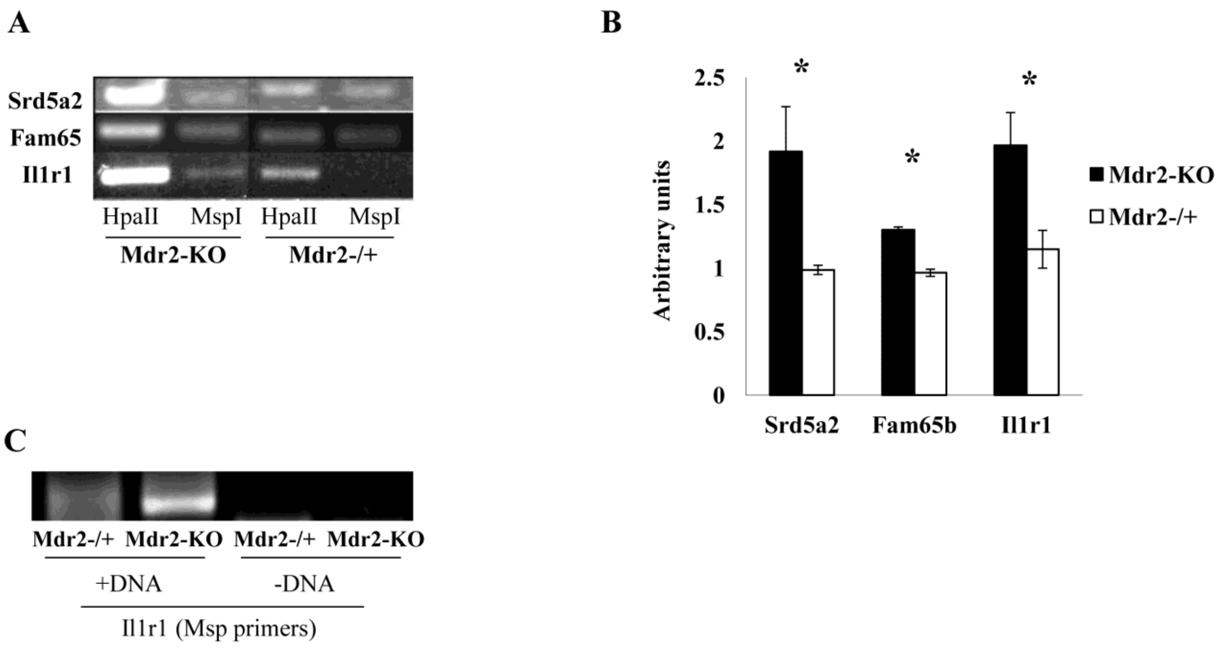

Supplementary Figure 4: Confirmation of the CGI microarray's results. 18 of the 20 tested genes were confirmed. A representative gel for Srd5a2, Fam65b and Il1r1 in 12-month-old mice is shown, as measured by MSRE-PCR after HpaII/MspI restriction (A) and quantified by ScionImage software (B), normalized to CryaA; three males per each experimental group; *$p<0.05$. (C) Confirmation of the Il1r1 gene hypermethylation by bisulfite Msp-PCR; 12-month-old Mdr2-KO liver; sample mix of 3 males per group.

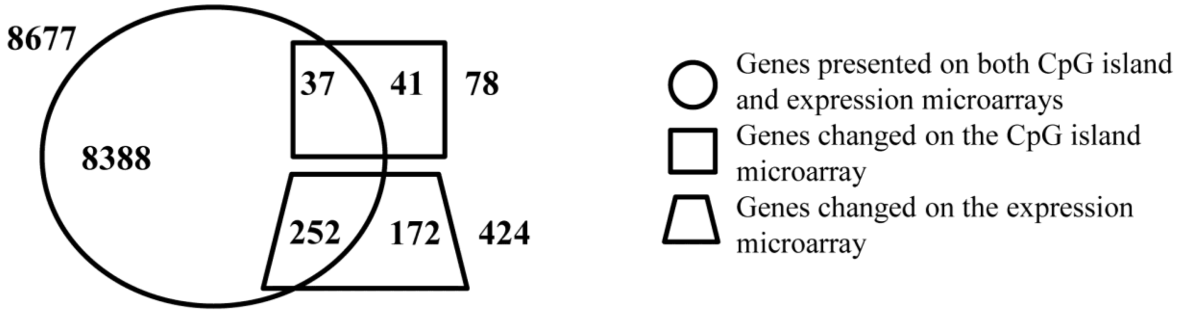

Supplementary Figure 5: Absence of an overlap between aberrantly methylated and aberrantly expressed genes. The Affymetrix gene expression and Agilent CpG island murine genome-scale microarrays had at least a 50% overlap: 8,677 common genes were represented on both arrays. 424 genes were aberrantly expressed (threshold > 1.9 fold; $p < 0.05$) and 78 genes were aberrantly methylated (Z score > 0.7) in the liver of Mdr2-KO compared to Mdr2$^{-/+}$ mice at the late precancerous stage (three 12-month-old males per group). None of the genes was simultaneously aberrantly methylated and expressed, as detected by microarrays.

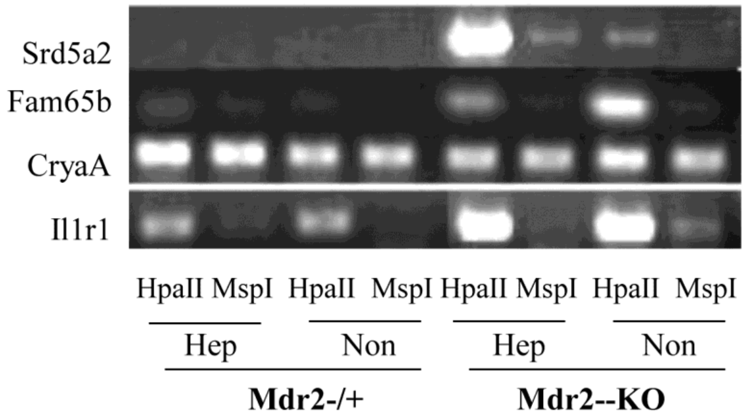

Supplementary Figure 6: Methylation of selected genes in liver cell fractions. Srd5a2 hypermethylated in Mdr2-KO hepatocytes (Hep) and Fam65b and Il1r1 hypermethylated in both Mdr2-KO hepatocyte (Hep) and non-hepatocyte fractions (Non). Representative gel of MSRE-PCR; quantification was performed by normalization to CryaA and relative to Mdr2$^{-/+}$, using the ScionImage software; three 12-month-old males per group. Significance – at $p < 0.05$.

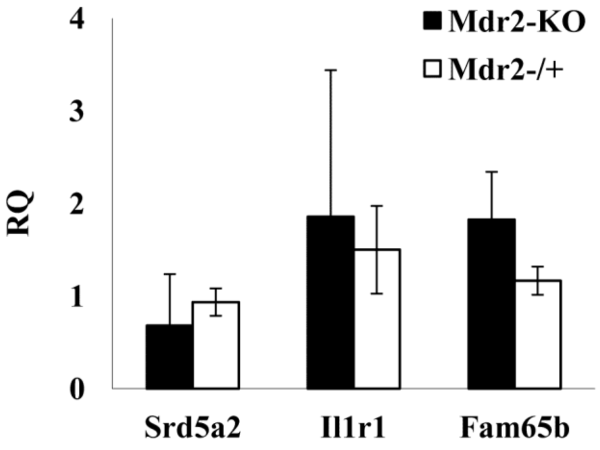

Supplementary Figure 7: Similar whole liver tissue expression of selected genes at the late precancerous stage. Srd5a2, Il1r1 and Fam65b genes expressed similarly in the livers of Mdr2-KO and $Mdr2^{-/+}$ 12-month-old mice. Real-time RT-PCR, normalized to Gapdh/Hprt; three 12-month-old males per group, $p > 0.05$.

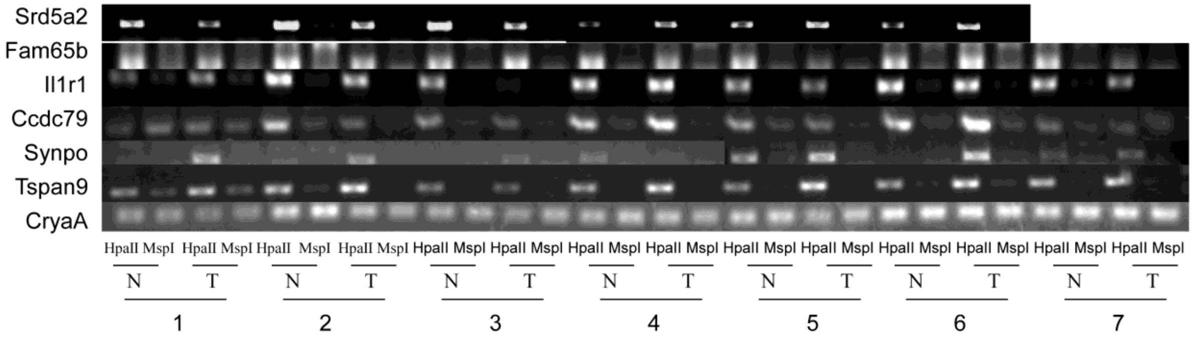

Supplementary Figure 8: Methylation status of selected genes in the non-tumor and tumor liver tissues of Mdr2-KO mice. Representative gels of MSRE-PCR. Methylation of the five selected genes was tested in seven HCC tumors (T) and their matched non-tumor (N) liver tissues of 16-month-old Mdr2-KO mice (cancerous stage). The selected genes were hypermethylated in the Mdr2-KO compared to Mdr2$^{-/+}$ non-tumor liver tissue at the late precancerous stage (12-month-old mice). Quantification was performed using the ScionImage software and normalization to *CryaA*; results are presented in Supplementary Table 5.

Supplementary Table 1: Chronic inflammation-induced pattern of CGIs' hypermethylation at the late precancerous stage. Arbitrary methylation levels, as determined using MeDIP followed by hybridization with CpG island microarray, of CGIs in the liver of Mdr2-KO and control Mdr2$^{-/+}$ mice (FVB/N, 12-month-old), in non-tumor (NT) and tumor (T) liver tissues of Mdr2-KO mice (FVB/N, 16-month-old), and in colon, brain, kidney, liver, lung, pancreas and spleen tissues of healthy C57BL/6 mice (3-month-old). Increasing levels of methylation: "-" (0% - 10%), "0" (11% – 69%), "+" (70% - 100%). Location of a CGI in a gene: "P" – promoter, "I" – inside gene body, "D" – downstream gene body, "U" - unknown. The first 30 CGIs of 30 genes were specifically hypermethylated in the Mdr2-KO liver at the late precancerous stage (12-month-old mice of the FVB/N strain) but mostly not in other murine tissues (3-month-old mice of the C57BL/6 strain).

CpG island in	Gene	Mdr2 -/+			Mdr2-KO precancer			NT	T	liver†	colon	brain	kidney	lung	muscle	pancreas	spleen	Intestine
P, I	Cdpf1	-	-	-	+	+	+	+	-	-	-	-	-	-	-	-	-	-
I	Adam11	-	-	-	+	+	+	+	+	-	-	-	-	-	-	-	-	-
I	Ccdc79	-	-	-	+	+	+	+	+	-	-	-	-	-	-	-	-	-
I	Fam65b	-	-	-	+	+	+	+	+	-	-	-	-	-	-	-	-	-
U	Klf14	-	-	-	+	+	+	+	+	-	-	-	-	-	-	-	-	-
I	Srd5a2	-	-	-	+	+	+	+	+	-	-	-	-	-	-	-	-	-
P, I	Bmp8b	-	-	-	+	+	+	+	+	-	-	-	0	-	-	-	-	-
I	Nfkb2	-	-	-	+	+	+	+	+	-	-	-	0	0	-	-	0	-
I	Mzf1	-	-	-	+	+	+	+	+	-	0	-	0	-	-	-	-	-
I	Fam196a	-	-	-	0	+	+	+	+	-	-	-	-	-	-	-	-	-
I	Pkdrej	-	-	-	0	+	+	+	0	-	-	-	-	-	-	-	-	-
U	Synpo	-	-	-	0	+	+	0	+	-	-	-	-	-	-	-	-	-
P	Fam131a	-	-	-	0	+	+	+	0	-	-	-	0	-	-	-	0	-
P, I	Fbrsl1	-	-	-	0	+	0	+	+	-	-	-	-	-	-	-	-	-
P, I	Tigd3	-	-	-	0	+	0	0	0	-	-	-	-	-	-	-	-	-
U	Tns1	-	-	-	0	+	0	-	+	-	-	-	-	-	-	-	-	-
I	Shcbp1l	-	-	-	0	+	0	-	0	-	0	-	-	-	-	-	-	-
U	Gm10190	-	-	-	0	+	0	0	+	-	0	-	-	0	-	-	-	0
U	Ddx43	-	-	-	0	0	0	0	+	-	-	-	-	-	-	-	-	-
P, I, D	Foxe1	-	-	-	0	0	0	0	+	-	-	-	-	-	-	-	-	-
P, I	Hmx1	-	-	-	0	0	0	0	+	-	-	-	-	-	-	-	-	-
I	Pcdh8	-	-	-	0	0	0	0	+	-	-	-	-	-	-	-	-	-
P, I	Psme4	-	-	-	0	0	0	0	+	-	-	-	-	-	-	-	-	-
I	Slc18a3	-	-	-	0	0	0	+	+	-	-	-	-	-	-	-	-	-

P	St8sia3	-	-	-	0	0	0	0	+	-	-	-	-	-	-	-	-	-
P	Tdrd5	-	-	-	0	0	0	-	+	-	-	-	-	-	-	-	-	-
P, I	Ephb3	-	-	-	0	0	0	0	+	-	-	-	0	-	-	-	-	-
I	Nckap5	-	-	-	0	0	0	+	+	-	-	-	0	-	-	-	-	-
P	Bcl9l	0	-	0	+	+	+	+	+	-	-	-	-	-	-	-	-	-
I	Trim14	0	-	0	+	+	+	+	+	-	-	0	-	-	-	-	-	-
I	Mapk8ip1	-	-	-	0	0	0	-	+	-	-	-	-	-	-	-	-	-
I	B3galt4	-	-	-	+	+	+	+	+	-	-	-	-	-	-	-	-	-
I	Tspan9	-	-	-	0	+	+	+	+	-	-	-	-	-	-	-	+	-
I	Fosb	-	-	-	0	+	+	+	+	-	-	-	-	+	-	-	-	-
I	Arhgap22	-	-	-	0	0	0	0	+	-	-	-	-	+	-	-	0	-
D	Cpeb3	-	-	-	0	+	+	+	0	-	-	-	0	0	-	-	-	-
U	Zfp518a	-	-	-	0	0	0	0	0	-	-	-	+	-	-	-	-	-
U	Gm70	-	-	-	+	+	+	+	+	-	-	-	+	0	-	-	-	-
P	Hoxa3	-	-	-	0	0	0	-	+	-	-	-	+	0	0	0	-	-
I	Kcnk12	-	-	-	0	+	0	0	+	-	-	-	+	0	+	-	0	-
I	Dnmbp	0	0	0	+	+	+	+	+	-	-	0	-	-	-	-	-	-
I	Nodal	0	-	+	+	+	+	+	+	-	-	0	-	-	-	-	-	-
P	Phldb2	+	+	+	-	-	-	-	-	-	-	+	-	-	-	-	-	-
P, I	Mmp23	-	-	-	0	0	0	0	+	-	0	-	-	-	-	-	-	-
P	Emp3	-	-	-	0	0	0	0	0	-	0	-	-	0	-	-	-	-
I	Ahdc1	-	-	-	0	+	0	0	+	-	0	-	0	-	-	-	-	-
P, I	Fam83f	-	-	-	0	0	0	0	+	-	0	-	+	0	-	-	0	-
I	Prrt3	-	-	-	0	+	+	0	+	-	0	-	+	0	-	-	0	0
I	Gpr44	-	-	-	0	+	+	+	+	-	0	-	+	0	0	-	-	0
P, I	Isyna1	0	0	0	+	+	+	+	+	-	0	-	+	0	0	-	0	-
I	Kctd1	-	-	-	0	+	+	+	+	-	0	-	+	+	0	-	+	0
P, I	Tcfl5	-	-	-	+	+	+	+	+	-	+	-	+	-	-	-	-	-
I	4921506M07Rik	-	-	-	0	+	+	+	+	-	+	-	+	0	0	-	0	0
I	Mex3a	-	-	-	0	+	0	+	+	-	+	-	+	0	0	-	+	+
P, I	2700086A05Rik	-	-	-	0	+	0	+	+	-	+	-	+	+	+	-	+	0
I	Sh3pxd2a	-	-	-	+	+	+	+	+	-	+	0	-	-	-	-	+	+
P, I	Zfp783	0	0	0	+	+	+	+	+	-	+	0	+	0	-	-	0	-
I	Rem2	-	-	-	0	0	0	0	0	0	-	-	-	-	-	-	-	-
I	Il1r1	-	-	-	+	+	+	+	+	0	-	-	-	-	-	-	-	0
I	Cyba	-	-	-	0	0	+	0	+	0	-	-	-	0	-	-	-	-
I	Foxp4	-	-	0	0	0	+	+	+	0	-	-	-	+	-	-	0	-
D	Otx1	-	-	-	0	+	0	0	+	0	-	-	0	-	-	-	-	-
I	Cyp4f39	-	-	-	0	0	0	+	+	0	-	-	0	0	-	-	-	-
D	Tspy12	-	-	-	0	0	0	+	0	0	0	-	-	0	-	-	-	-
I	Pcdha3	-	-	-	0	?	0	+	+	0	0	-	+	0	0	0	0	+
P	Pcdhgb2	-	-	-	0	+	+	+	0	0	0	-	+	+	+	-	0	+
I	Celsr1	-	-	-	+	+	+	+	+	0	0	0	+	+	0	-	+	-
I	C2cd4c	0	0	0	+	+	+	+	+	0	+	0	-	-	-	0	-	-
I	Cyp46a1	0	0	+	-	-	-	-	0	+	-	-	-	-	-	-	0	-

P	Zfp3611b (non-mus)	-	-	-	0	0	0	0	0	+	-	+	+	+	+	-	+	-
I	Bmi1	-	-	-	+	0	+	0	0	+	0	0	-	+	-	-	+	+
I	Dysf	-	-	-	+	+	+	+	0	+	0	0	+	+	+	+	+	+
P, I	Praf2	-	-	-	0	+	0	0	0	+	0	+	+	+	+	-	+	-
I	Gm996	-	-	-	0	0	0	0	+	+	+	-	+	0	-	-	0	+
D	Mir33	0	-	-	+	+	+	+	+	+	+	-	+	+	+	-	+	+

* similar in 12-month-old C57Bl/6 mice
† similar in 12- and 18-month-old C57Bl/6 mice

Supplementary Table 2: Four patterns of DNA demethylation in Mdr2-KO compared to Mdr2$^{-/+}$ liver at 9 and 12 months of age. Gene symbols of the selected genes which comprise four groups based on different age-dependent methylation patterns (see Fig. 3) in Mdr2-KO compared to Mdr2$^{-/+}$ liver at the precancerous stages (9- and 12-month-old mice).

Methylation pattern	Genes (#)	Gene symbols
A	11	Adam11, Fam65b, Fosb, Il1r1, Mzf1, Nfkb2, Otx1, Synpo, Tcfl5, Tns1, Tspan9
B	4	Ephb3, Pcdh8, Psme4, Srd5a2
C	2	Bmp8b, Fam196a
D	1	Ccdc79

Supplementary Table 3: **Expression of selected aberrantly methylated genes in the whole liver gene at the late precancerous stage**. Relative expression (Mdr2-KO compared to Mdr2$^{-/+}$ liver) of the selected genes at the late precancerous stage (12-month-old mice) tested by RT-PCR. "Up" – upregulated, "Down" – downregulated in the Mdr2-KO compared to Mdr2$^{-/+}$ liver. At least three males per experimental group. Most represented genes were hypermethylated, while Lrrc16a and Phldb2 were hypomethylated in the Mdr2-KO compared to Mdr2$^{-/+}$ liver at this age.

Methylation pattern	Total	Expressed in the liver			Not expressed
		Up	Down	Unchanged	
Real-Time PCR	6	1	0	4	1
		Bmp8b		Adam11, Il1r1, Klf14, Srd5a2	Fam65b
sqRT-PCR	12	3	1	3	5
		Cyba, Mmp23, Synpo	Lrrc16a	Ethe1, Phldb2, St8sia3	Ccdc79, Foxe1, Gm70, Slc18a3, Tcfl5

Supplementary Table 4: **Comparative methylation of selected genes in the tumor and matched non-tumor liver tissues of Mdr2-KO mice.** Methylation levels of the five selected genes were tested in seven HCC tumors (T) and their matched non-tumor (N) liver tissues of 16-month-old Mdr2-KO mice (cancerous stage). Numbers of tumors with preferential methylation of non-tumor (N>T) or tumor (T>N) liver tissue are shown. The selected genes were hypermethylated in the Mdr2-KO compared to Mdr2$^{-/+}$ non-tumor liver tissue at the late precancerous stage (12-month-old mice).

**Genes**	**N > T**	**T > N**	**N = T**
Srd5a2	3	3	-
Fam65b	5	2	-
Il1r1	5	1	1
Synpo	1	5	1
Tspan9	1	5	1

Supplementary Table 5: PCR primers used in this study. Primers for expression assay were chosen from different exons, primers for MSRE include CCGG restriction site. All primers are of mouse origin. Excluding primers for the Tet genes and for Taqman and SYBR RT-PCR assays, all other primers were designed using the Primer3 software [1]. Primers for quantitative RT-PCR of the Tet1 and Tet2 genes are described in [2].

Gene symbol	Forward primer	Reverse primer	PCR method*	Product size	PCR assay
Adam11	CGGAAACGGCTTCGTGGAGG	CGGTCCCGGGTTTTACAGCG	SYBR	315	gene expression
Bcl9l	CGCCCAGTCTGCCCAACCGG	GCGAGGCAGCGAGCGAGGTT	SQ	140	gene expression
Cyba	GGTATTTCGGCGCCTAC	ACTTCTGTCCACATCGC	SQ	108	gene expression
Fam65b	GGGCCCAATGGCATCATCCG	AAGGCCCGGTAGACCTCCTC	SYBR	165	gene expression
Foxe1	TTCGGCTTGGTTCCCGAGCG	TCCGAGTTGCCCACCGGGAT	SQ	378	gene expression
Gm70	GCGTGGCGGTATCCCAGGTG	CCTGGAGGGACAGCAGGGCA	SQ	392	gene expression
Otx1	CCCCCATACGGCATGAACGG	CGGCCGGGTTTTCGTTCCATT	SYBR	300	gene expression
Pcdh8	TGGACCGTGAGCGTCTGTGT	GAGGCTCGGCTAGGCGTACA	SYBR	264	gene expression
Synpo	GGCCCTCCAAACAGGGCGTC	CAGCGGTGGGCTGACTGTGG	SYBR	422	gene expression
Tspyl2	AGGCTCTGGATAACCGCACCA	AAGCTTTTGGGACCGGCAGC	SYBR	213	gene expression
Cbs	CTCACATCCTGGAGATGGAC	GGTGTCTCTGAAAGCCAAGA	SQ	255	gene expression
Cxcl14	ACGGGTCCAAGTGTAAGTGT	GCAAAGTCCTCTGCTGAAGT	SQ	301	gene expression
Dgkz	CAGATCAGCCCCTGGCATGGAGAC	CCCACCCCGCAGCTGGGAAA	SQ	213	gene expression
Egfr	TCCTGGAGGGGGAACCAAGGGA	GGCCAATCCCAAGGGCCACCA	SQ	381	gene expression
Bmp8b	MM03033968_m1		TQ		gene expression
Il1r1	MM00434237_m1		TQ		gene expression
Srd5a2	MM00446421_m1		TQ		gene expression
Klf14	MM03646643_s1		TQ		gene expression
Ube2a	MM00499179_m1		TQ		gene expression
Mmp23	CGGTACAGGGCCGCTGGTTG	GTGTCAGCGTGTAGCGGCGT	SQ	210	gene expression
Synpo	GGCCCTCCAAACAGGGCGTC	CAGCGGTGGGCTGACTGTGG	SQ	422	gene expression
Lrrc16a	GGTGAGTAGGTCCAATCGAC	CAGGCCCAAGAGCAGCG	SQ	623	gene expression
Stx11	GTTCGGGGTTGGCTGGAG	CTCTGCAAGCCGATCCTTC	SQ	217	gene expression
Pim1	GATCATCAAGGGCCAAGTGT	GATGGTTCCGGATTTCTTCA	SQ	122	gene expression
Ethe	ACTGTCTGATCTACCCTGCTCA	TTGACAAACTCCTCACAGCTGA	SQ	823	gene expression
Mbd2	CCGACATCCTGTCCCGGGCT	GCTGCACTGCACCGGAAGGG	SQ	328	gene expression
Mmp14	CACGCCACTGCGCTTCCGAG	GTTGGGCCCATAGGCGGGGT	SQ	490	gene expression
Mtss1	TGGGTGCAGGCCCTTTCCCT	GGCTTTGCCCAGTCCTTCCAGC	SQ	154	gene expression
Pdk4	GCCAGCCTAGGTGGGCGTCA	CCGTGGCCCTCATGGCATTCTTG	SQ	812	gene expression
Stmn1	CGGACCGAGCAGGGCTTTCCTT	GCCATCTGCGCCTCCCGGTT	SQ	422	gene expression
Arl6ip1	GTGTTCGCTCGTTGATAACCG	CCCATCGAAGGACTTTGTCAG	SQ, SYBR	156	gene expression
Arl6ip1	MM01274631_m1				gene expression
Hprt	GTTAAGCAGTACAGCCCCAAA	AGGGCATATCCAACAACAAACTT	SQ, SYBR	130	gene expression

Hprt	MM00446968_m1		TQ		gene expression
mtD1	CCACCAACAGCTACCATTAC	TGGGTTTATGAGGTCTGGGT	SQ	747	mitochondrial DNA
mtDNA	GCTCTACCTCACCATCTCTTG	CCAGTATGCTTACCTTGTTACG	SQ	360	mitochondrial DNA
Ccdc79	CCCAGCCTCCGGGTCCACCG	CGGGAGGCGTGCATGGTGCC	SQ	172	MSRE
Ephb3	GTGCCCCGAGAACCTGCGAC	GATGCCGGCAGGTCGTTCCC	SQ	127	MSRE
Fam65b	AGCGCTGGGGCCACGACTGT	ACCACCTCGGCACCCACGCA	SQ	122	MSRE
Fosb	GCTGCTCGCTGCCGCTGGTG	AGCAGCCGAGACGCACCCCC	SQ	175	MSRE
Mzf1	TGGATGGCGCTGGACCAGGC	GGCCTTTCGTGTGCCGCGAC	SQ	186	MSRE
Nfkb2	CGGGACACCGATGCTGGCGA	TCCCGCTAGCAGAGCACGCG	SQ	275	MSRE
Otx1	GCGAGCGGACAGACACGGGC	CCGCCGAGCACGCCTGCAAC	SQ	369	MSRE
Pcdh8	ACGTCTCGGATGCGCACGAT	GCAGGTGGACTACGAGCGCC	SQ	111	MSRE
Srd5a2	AACCAGGCTATGCGTGCGGG	GGCGCTCCATAAAGGGGCCC	SQ	230	MSRE
Synpo	CTGCGCAGGTGGCAAGGGCGA	GGCTCCCGCTTGGGTCCCCTTCA	SQ	240	MSRE
Tspan9	GGTGCCGTAGAAGGCGCCAG	GCTCAGAACCCGTGCCCGAC	SQ	230	MSRE
Adam11	AAACGGCTTCGTGGAGGCGG	CTCACCTTGCAGCGGCGACA	SQ	265	MSRE
Il1r1	AGCGCGGCGCCCACCTAGAA	CGGGTGCCCCAGTGGCGATG	SQ	186	MSRE
Tcfl5	CGCACTTGTTGAGCGCCACG	GACCGTACGCGTCCGTCTGG	SQ	107	MSRE
Tns1	CCGCACCCGCTCACCTTTGC	CGCGCTGACTCCGGGAAGGA	SQ	251	MSRE
Psme4	GCGGCAGCGATGGCGATGAT	CTGGGCTTGCGGCCTCTCAC	SQ	133	MSRE
Bmp8b	AGGAGGCGTCTTCCCCGGAC	GGCGGATGCCAACCATGCCT	SQ	121	MSRE
Fam196a	ACACTGGGCTACACCCGCCG	CGCGGCAAGACATGCCCGAG	SQ	211	MSRE
CryaA	CATTCAGCATCCTTGGTTCA	GCAGCAGGTCGTACTCAAAA	SQ, SYBR	101	MSRE & MeDIP; control gene
Aprt	GGGATATCTCGCCCCTCTT	CACTCGCCTGCGATGTAGT	SYBR	115	MeDIP, control gene
B1	TAACCTCAAACTCAAAAATCCACC	GTTGGGTGTAGTGGTATATATTTTT AATTTTA	SQ	82	Unmethylated, SINE
B1	CTCGAACTCAAAAATCCGCC	GTCGGGCGTAGTGGTATATATTTTT	SQ	78	Methylated, SINE
Alb	GAGACCTTCACCTTCCACTC	GGTTGTGGTTGTGATGTGTT	SQ	262	gene expression
Tcr	ACCCAGAACCTGCTGTGTA	ATTCGGAGTCCCATAACTGA	SQ	343	gene expression
Tet1	GCTGGATTGAAGGAACAGGA	GTCTCCATGAGCTCCCTGAC	SYBR	128	gene expression
Tet2	GTCAACAGGACATGATCCAGGAG	CCTGTTCCATCAGGCTTGCT	SYBR	103	gene expression

- SYBR – real-time PCR with SYBR mix; TQ - real-time PCR with Taqman primers; SQ- semi-quantitative PCR.

References

1. Rozen S and Skaletsky HJ, *Primer3 on the WWW for general users and for biologist programmers*, in *Bioinformatics Methods and Protocols: Methods in Molecular Biology*, S. Krawetz and S. Misener, Editors. 2000, Humana Press: Totowa, NJ. p. 365-386.
2. Dawlaty MM, Breiling A, Le T, Raddatz G, Barrasa MI, Cheng AW, Gao Q, Powell BE, Li Z, Xu M, Faull KF, Lyko F and Jaenisch R. Combined deficiency of Tet1 and Tet2 causes epigenetic abnormalities but is compatible with postnatal development. Dev Cell. 2013; 24(3):310-323.
